# Inhibition of HSP90 Preserves Blood–Brain Barrier Integrity after Cortical Spreading Depression

**DOI:** 10.3390/pharmaceutics14081665

**Published:** 2022-08-10

**Authors:** Seph M. Palomino, Aidan A. Levine, Jared Wahl, Erika Liktor-Busa, John M. Streicher, Tally M. Largent-Milnes

**Affiliations:** Department of Pharmacology, University of Arizona, 1501 N. Campbell Avenue, Tucson, AZ 85719, USA

**Keywords:** heat shock protein 90, cortical spreading depression, blood–brain barrier, 17-AAG, blood–endothelial barrier, in situ brain perfusion

## Abstract

Cortical spreading depression (CSD) is a pathophysiological mechanism underlying headache disorders, including migraine. Blood–brain barrier (BBB) permeability is increased during CSD. Recent papers have suggested that heat shock proteins (HSP) contribute to the integrity of the blood–brain barrier. In this study, the possible role of HSP90 in CSD-associated blood–brain barrier leak at the endothelial cell was investigated using an in vitro model, for the blood–endothelial barrier (BEB), and an in vivo model with an intact BBB. We measured barrier integrity using trans endothelial electric resistance (TEER) across a monolayer of rodent brain endothelial cells (bEnd.3), a sucrose uptake assay, and in situ brain perfusion using female Sprague Dawley rats. CSD was induced by application of 60 mM KCl for 5 min in in vitro experiments or cortical injection of KCl (1 M, 0.5 µL) through a dural cannula in vivo. HSP90 was selectively blocked by 17-AAG. Our data showed that preincubation with 17-AAG (1 µM) prevented the reduction of TEER values caused by the KCl pulse on the monolayer of bEnd.3 cells. The elevated uptake of ^14^C-sucrose across the same endothelial monolayer induced by the KCl pulse was significantly reduced after preincubation with HSP90 inhibitor. Pre-exposure to 17-AAG significantly mitigated the transient BBB leak after CSD induced by cortical KCl injection as determined by in situ brain perfusion in female rats. Our results demonstrated that inhibition of HSP90 with the selective agent 17-AAG reduced CSD-associated BEB/BBB paracellular leak. Overall, this novel observation supports HSP90 inhibition mitigates KCl-induced BBB permeability and suggests the development of new therapeutic approaches targeting HSP90 in headache disorders.

## 1. Introduction

Heat shock protein 90 (HSP90) is a central regulator of protein homeostasis under physiological and pathogenic conditions. This molecular chaperone facilitates maturation of its substrates, and plays a part in proper protein folding and trafficking [1]. Its substrates or client proteins include kinases, transcription factors, steroid hormone receptors, and E3 ubiquitin ligases [1]. HSP90 is ubiquitously expressed throughout the body, comprising 2–3% of the total protein content of the cell under physiological conditions [2]. There are also four isoforms present in different compartments of the cell [2]. The chaperone is a homodimer which will bind with substrates based on a conformational change which is dependent upon ATP hydrolysis [1]. To date, there has been extensive research into HSP90 in the field of cancer since it is upregulated and can lead to increased cancer cell proliferation which can cause a higher chance of metastasis [2]. Recently, HSP90 has been studied in the context of pain, specifically with opioid signaling [2,3]; moreover, the role of HSP90 in other diseases, such as Alzheimer’s and rheumatoid arthritis, has also been explored. Currently, there are several generations of HSP90 inhibitors which have undergone clinical trials, and these compounds can be repurposed for use in other clinical situations [2,3,4].

HSP chaperones have been proposed to play a role in BBB integrity [5]. Although publications mainly report protective effects of HSPs in certain types of neurodegenerative diseases, such as Parkinson’s and Huntington’s disease, contrasting results were also obtained [6]. Increased expression of HSP70 and 90 are linked to impaired BBB integrity and/or neuroinflammation in autism spectrum disorders, cancers (e.g., glioblastoma, meningioma), amyotrophic lateral sclerosis (ALS), Alzheimer’s disease (AD), intracerebral hemorrhage, and stroke [5].

Primary and secondary headache disorders, such as migraine and medication overuse headache, or stroke and traumatic brain injury, have been associated with a pathophysiological phenomenon termed cortical spreading depression (CSD) [7]. Mechanistically, CSD events are driven by increases in extracellular K^+^ that induce depolarizing waves of activity spreading across the cortex, followed by a prolonged period of suppressed activity (i.e., depression) [8]. Direct cortical dysfunction, including breakdown of ionic gradients, leads to neuronal, glial, and vascular activation [9]. Moreover, transient opening of the blood–brain barrier (BBB) to small molecules in the cortex, but not the brainstem, has been reported during CSD events, resulting from changes to cerebral blood flow and dysregulated neurovascular coupling [10,11]. Given the overlap between the neurological diseases mentioned above and the reduced blood–brain barrier integrity caused by CSD events, we asked whether HSP90 plays a role in CSD-induced transient paracellular leak at the BBB. In this study, pharmacological inhibition of HSP90 by application of 17-AAG was utilized in combination with in vitro and in vivo approaches to answer this scientific question.

## 2. Materials and Methods

### 2.1. Cell Culture

The bEnd.3 (CRL-2299, ATCC) cell line was used for in vitro experiments, cultured as described in Liktor-Busa et al. [12]. Briefly, the endothelial cells were maintained in DMEM (Gibco, 11995–065, Waltham, MA, USA), supplemented with 2 mM L-glutamine (ThermoScientific, 25030081, Waltham, MA, USA), 10% fetal bovine serum (Gibco, 10082139), and penicillin (100 UI/mL)-streptomycin (100 μg/mL) (Invitrogen, 15140122, Waltham, MA, USA). The media of bEnd.3 cells were changed to astrocyte-conditioned media (ACM) harvested from confluent C8-D1A flasks 24 h before treatment. The C8-D1A cells were cultured in DMEM supplemented with 10% fetal bovine serum, and penicillin-streptomycin. Cell lines were maintained at 37 °C in a humidified 5% CO_2_/95% air atmosphere. Subsequently, bEnd.3 cells were treated with 60 mM KCl for 5 min, which is a typical condition to evoke potassium-triggered spreading depolarization in live brain slices [13]. Then, 10 mM 17-AAG (Fisher Biosciences, Hampton, NH, USA) was made up in 100% DMSO then diluted in DMEM to 1 µM concentration and applied apically to bEnd.3 cells for 24 h prior to KCl exposure. This dose and incubation time were established in our previous work [14].

### 2.2. Proteomic Analysis

bEnd.3 cells were treated with either artificial cerebrospinal fluid (aCSF) or KCl (60 mM) for 5 min in 6-well plates, then lysed in lysis buffer (20 mM Tris-HCl (pH 7.4), 50 mM NaCl, 2 mM MgCl_2_ hexahydrate, 1% (*v*/*v*) NP40, 0.5% (*w*/*v*) sodium deoxycholate, 0.1% (*w*/*v*) SDS supplemented with protease and phosphatase inhibitor cocktail (BiMake, Houston, TX, USA)). The cell lysate was centrifuged at 12,000× *g* for 10 min at 4 °C. The supernatant was collected and used for the subsequent analysis as we have previously reported [15]. The protein content was determined with BCA assay. Here, 200 μg of harvested cell lysate supernatant was separated on a 10% SDS-PAGE gel and stained for total protein with Bio-Safe Coomassie G-250 Stain. Lanes from the gel were separated, cut into six slices, and underwent trypsin digestion; the resulting peptides were purified by C18 desalting performed as described [16].

High-performance liquid chromatography–electrospray ionization tandem mass spectrometry (HPLC-ESI-MS/MS) was performed in positive ion mode on an Orbitrap Fusion Lumos tribrid mass spectrometer (ThermoScientific, Waltham, MA, USA) fitted with an EASY-spray source (Thermo). NanoLC was performed according to protocol published by Kruse et al. [16]. Tandem mass spectra were extracted from files in Xcalibur ‘RAW’ and ProteoWizard 3.0 msConvert script was used to assign charge states with default parameters. Mascot (Matrix Science, ver 2.6.0, Boston, MA, USA) software was used with default probability cut off score settings to search fragment mass spectra against the *Mus musculus* database in SwissProt_2018_01 (16965 entries). The search variables used were as follows: 10 ppm mass tolerance for precursor ion masses and 0.5 Da for product ion masses, trypsin digestion, maxima of two missed tryptic cleavages, variable modifications of phosphorylation of threonine, tyrosine, and serine, and oxidation of methionine. Scaffold software (Proteome Software, ver 4.8.7, Portland, OR, USA) was used to cross correlate Mascot search results with X! Tandem software. Significance value was set at *p* ≥ 0.05. Targeted evaluation for Hsp90, 70, and co-chaperones was performed.

Ion intensity-based label free quantification was performed using Progenesis QI for proteomics software (Nonlinear Dynamics, ver 2.4, Waters Corporation, Milford, MA, USA). Raw files were imported and converted into two-dimensional maps with the *y* axis defined as time, and the *x* axis defined as *m*/*z*, which was then followed by selection of a reference run for alignment. The aligned runs were then used to create an aggregate data set containing all peak information from all samples, after which the data pool was narrowed down to only +2, +3, and +4 charged ions for further analyses. The top 8 most intense precursors of a given feature were grouped into a peak list of fragment ion spectra and exported in a Mascot generic file (.mgf) and searched against the *Mus musculus* SwissProt_2018_01 database utilizing Mascot software. The following search variables were used: 10 ppm mass tolerance for precursor ion masses, and 0.5 Da for product ion masses, trypsin digestion, maxima of two missed tryptic cleavages, variable modifications of oxidation of methionine and phosphorylation of serine, tyrosine, and threonine, 13C = 1. The data was collected into a Mascot.xml file and imported into Progenesis allowing for assignment of peptides and proteins. Peptides with a Mascot ion score < 25 were not used for further analyses. Non-conflicting peptides and precursor ion abundance values were normalized using a reference run to perform protein quantification.

### 2.3. Trans Endothelial Electrical Resistance (TEER)

TEER is an established method of evaluating endothelial cell barrier integrity which captures active and passive breaches in vitro [17]. TEER was assessed via the two-electrode chopstick method (EVOM2), as described in Blawn et al. [15]. Briefly, bEnd.3 cells were seeded on collagen-coated transwell inserts (Corning) at 6.0 × 10^4^ cells/cm^2^ density. Then, 500 uL of bEnd.3 medium was used on the luminal side, and 1000 uL of astro-conditioned media (ACM) was added to the abluminal side to create an in vitro model of the blood–brain barrier. ACM was collected as described above. Twenty-four hours before the KCl pulse, the cells were treated with either 17-AAG (1 µM) or vehicle (1% DMSO in media). On the day of the experiment, the media on the abluminal side was changed to media containing 60 mM KCl (KCl pulse). Media containing aCSF were utilized as vehicle control. Baseline measurements were taken before any treatment and right before the KCl pulse (0 time-point). After 5 min, pulse media were removed and replaced with fresh bEnd.3 media. The TEER measurements were performed immediately post-pulse (5 min time-point), then 10, 20, 30, 60, 120, and 180 min after the KCl pulse. Time-points were chosen based on former data indicating rapid, but temporal changes in the blood–brain barrier integrity after any insult. All measurements were repeated in triplicate over three individual experiments in a temperature-controlled environment (at 37 °C). Blank wells have a TEER of 93.1 mohm/cm^2^.

### 2.4. Sucrose Transport

Functional implications of monolayer integrity or lack thereof were assessed in vitro as luminal to abluminal transport/uptake of ^14^C-sucrose. A detailed description of uptake experiments is published by Liktor-Busa et al. [12]. Briefly, bEnd.3 cells were seeded on the luminal side of collagen-coated filter membranes of 24-well inserts (Costar, Corning, NY, USA). The cells were cultured as described in Section 2.3. On the day of the experiment, 60 mM KCl in bEnd.3 medium was used for 5 min (KCl pulse) on the abluminal side. Media with aCSF were applied as vehicle control. ^14^C-sucrose (PerkinElmer, NEC100XOO1MC, Waltham, MA, USA) was added to the luminal side at 0.25 μCi/mL concentration to monitor the paracellular uptake. Radioligand was added when KCl or aCSF was applied on the abluminal side. The radioactivity of samples harvested from the abluminal side at 5 and 30 min was measured using a 1450 LSC and Luminescence Counter (PerkinElmer). All experiments were carried out over three independent experiments each, using the 3–4 transwell inserts/condition.

### 2.5. In Vivo Experiments—Dural Cannulation and In Situ Brain Perfusion

Intact, female Sprague Dawley rats (200–250 g) were purchased from Envigo (Indianapolis, IN, USA) and housed in a climate-controlled room on a regular 12/12 h light/dark cycle with lights on at 7:00 am with food and water available ad libitum. All procedures were performed during the 12 h light cycle and according to the policies and recommendations of the International Association for the Study of Pain and the NIH guidelines for laboratory animals, and with IACUC approval from the University of Arizona (17-223). Dural cannulation and in situ brain perfusion were performed as previously described by Cottier et al. and Liktor-Busa et al. [11,12].

Briefly, 45:5:2 mg/kg cocktail of ketamine/xylazine/acepromazine was intraperitoneally (IP) administered to induce anesthesia. Rats were placed in a stereotactic frame (Stoelting Co., Ltd., Dale, IL, USA) and a 1.5 to 2 cm incision was made to expose the skull. A 0.66 to 1 mm hole (Pinprick/KCl: −6 mm A/P, −3 mm M/L from bregma) was made with a hand drill (DH-0 Pin Vise; Plastics One) to carefully expose, but not damage, the dura. A guide cannula (0.5 mm from top of skull, 22 GA, #C313G; Plastics One) was inserted into the hole and sealed into place with glue. Two additional 1 mm holes were made caudal to the cannula to receive stainless-steel screws (#MPX-080-3F-1M; Small Parts), and dental acrylic was used to fix the cannula to the screws. A dummy cannula (#C313DC; Plastics One) was inserted to ensure patency of the guide cannula. Rats were housed individually and allowed 1 week to recover. Cannula placement and dural integrity at screw placement were confirmed postmortem. After the recovery period, CSD was induced by delivery of a focal injection of 0.5 µL of 1 M KCl through the guide cannula using a Hamilton injector (30 GA, #80308 701 SN, Hamilton Company, Reno, NV, USA). Injection of aCSF into the cerebral cortex was used as a vehicle control. aCSF comprised 145 mM NaCl, 2.7 mM KCl, 1 mM MgCl_2_, 1.2 mM CaCl_2_, and 2 mM Na_2_HPO_4_ (pH 7.4). Cortical injections were performed 24 h after dural application of 5 μL of vehicle (1% DMSO in aCSF) or 17-AAG (0.5 nmol) [15]. This dose and treatment time for 17-AAG in rodents was also established in our previous work [14].

For in situ brain perfusion, rats were anesthetized as described above and heparinized (10,000 U/kg, i.p.). Body temperature was maintained at 37 °C using a heating pad. The common carotid arteries were bi-laterally cannulated and connected to a perfusion circuit. The perfusate was an erythrocyte-free modified mammalian Ringer’s solution: 117 mM NaCl, 4.7 mM KCl, 0.8 mM MgSO_4_, 1.2 mM KH_2_PO_4_, 2.5 mM CaCl_2_, 10 mM D-glucose, 3.9% (*w*/*v*) dextran (MW 60,000), and 1.0 g/L bovine serum albumin (Type IV), pH 7.4, warmed to 37 °C and oxygenated with 95% O_2_/5% CO_2_, and Evan’s blue dye (55 mg/L) was added to the perfusate to serve as a visual marker of BBB integrity. Perfusion pressure and flow rate were maintained at 95–105 mmHg and 3.1 mL/min, respectively. Both jugular veins were cut to allow for drainage of the perfusate. Using a slow-drive syringe pump (0.5 mL/min per hemisphere; Harvard Apparatus, Holliston, MA, USA), ^14^C-sucrose (0.5 μCi/mL) was added to the inflowing perfusate for 10 min followed by a 2 min washout period in which non-radioactive Ringer’s solution was perfused to clear vascular radioactivity. After perfusion, the rat was decapitated, and the brain was harvested. The meninges and choroid plexus were removed, cerebral hemispheres were sectioned, and the brain was divided and placed into pre-weighed vials. One milliliter of TS2 tissue solubilizer was added to each vial, and the samples were solubilized for 48 h at room temperature. To eliminate chemiluminescence, 100 μL of 30% glacial acetic acid was added, along with 1.5 mL Optiphase SuperMix liquid scintillation cocktail (PerkinElmer). Perfusion media were also sampled and placed in triplicate 100 μL aliquots in scintillation vials and processed in the same manner as the tissue samples. All samples were then measured for disintegrations per minute (dpm; 1450 LSC and Luminescence Counter; PerkinElmer). The ratio of the concentration of ^14^C-sucrose in tissue (C_brain_; in dpm/g) was compared with perfusate (C_perfusate_; in dpm/mL) and expressed as a percentage ratio (RBR) R_brain region_ = (C_brain region_/C_perfusate_) × 100%.

### 2.6. Western Blot

For Western immunoblotting, rats were injected with 17-AAG (0.5 nmol) or vehicle (1% DMSO in saline) through dural cannula 24 h before cortical KCl application. CSD was induced with focal injection of 0.5 µL of 1 M KCl. Ninety minutes after cortical KCl injection, tissue was collected as described in Liktor-Busa et al. [12]. Briefly, rats were anesthetized with ketamine/xylazine mix (80:10 mg/kg, i.p.), then transcardially perfused with ice cold 0.1 M phosphate buffer at flow rates to not burst microvasculature (i.e., 3.1 mL/min). After decapitation, periaqueductal grey (PAG) was dissected, flash frozen in liquid nitrogen, and stored at −80 °C until further use. On the day of the experiment, tissue samples were thawed on ice, Dounce homogenized in ice cold lysis buffer (20 mM Tris-HCl (pH 7.4), 50 mM NaCl, 2 mM MgCl_2_ hexahydrate, 1% (*v*/*v*) NP40, 0.5% (*w*/*v*) sodium deoxycholate, 0.1% (*w*/*v*) SDS supplemented with protease and phosphatase inhibitor cocktail (BiMake)). The tissue lysate was centrifuged at 12,000× *g* for 10 min at 4 °C, then the supernatant was collected and used for determination of protein content with a BCA assay. Total protein (40 μg) from tissue supernatant was loaded into TGX precast gels (4–20% CriterionTM, BioRad) and transferred to nitrocellulose membrane (AmershamTM ProtranTM, GE Healthcare). After transfer, the membrane was blocked at room temperature for 1 h in blocking buffer (5% dry milk in Tris-buffered saline with tween 20 (TBST)). The following primary antibodies were diluted in blocking buffer (5% BSA in TBST): Claudin-5 (ThermoFisher Scientific, Waltham, MA, USA, 35-2500, 1:500), and α-tubulin (Cell Signaling, Danvers, MA, USA, 3873S, 1:10,000). The membrane was incubated in diluted primary antibodies for 48 h at 4 °C. The membrane was washed three times in TBST for 5 min each then incubated with Goat αMouse IRDye-680 (LiCor, 926–68072, Lincoln, NE, USA) in 5% milk in TBST for 1 h rocking at room temperature. The membrane was washed again three times for five minutes each and imaged with an Azure Sapphire laser imager (Azure Biosystems, Dublin, CA, USA). The Western image was produced using Azure Capture (AC1078) software and analyzed using Azure Spot Western Analysis software. Bands were quantified using ImageJ FIJI software (ImageJ 1.53s, NIH) open access from NIH.

### 2.7. Statistics

GraphPad Prism 7.0 and 8.3.1 software (GraphPad Software, San Diego, CA, USA) were used for statistical analysis; numbers needed for each experiment were determined using G.Power3.1 for 80% power to detect a 20% difference when alpha = 0.05. Unless otherwise stated, the data were expressed as mean ± SEM or SD as appropriate. Groups were compared by unpaired *t*-test or one-way ANOVA with Tukey’s post-test, as indicated. Differences were considered significant if *p* ≤ 0.05.

## 3. Results and Discussion

### 3.1. CSD-Like Environments Increase HSP90a Expression in Brain Endothelial Cells In Vitro

**Figure 1:**
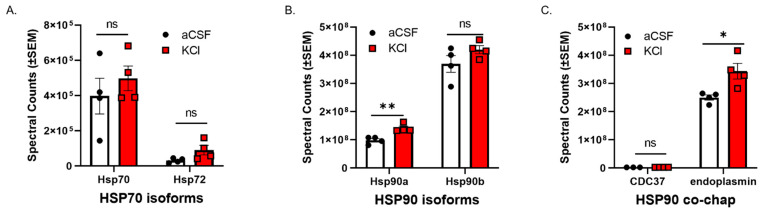
**KCl stimulation increases the expression of HSP90a and the co-chaperone endoplasmin in murine brain endothelial cells**. bEnd.3 mouse endothelial cells cultured in the presence of astrocyte-conditioned media were treated with 60 mM KCl or aCSF for 5 min. The KCl pulse (60 mM) mimicked the conditions during cortical spreading depression. aCSF was used as vehicle control. The cells were then lysed in lysis buffer and subjected to proteomic analysis. The graphs show proteomic spectral counts for HSP70 isoforms (**A**), HSP90 isoforms (**B**), and HSP90 co-chaperones (**C**) detected by unlabeled proteomic approaches. (**A**) No significant differences were observed in the expression of HSP70 isoforms between KCl vs. aCSF-treated cells. (**B**) The KCl pulse significantly increased the expression of HSP90a, but not HSP90b compared to aCSF control. (**C**) Significant increase in the expression of endoplasmin was detected in KCl-treated cells, compared to aCSF ones, but there was no significant difference in the expression of CDC37 between KCl and aCSF-treated cells. ns = non-significant, * *p* < 0.05, ** *p* < 0.01, as assessed by two-way ANOVA, Sidak–Holms corrected *t*-test. Values are mean ± SEM (*n* = 4). Circles and squares indicate individual subjects.

CSD events are reported to induce a transient increase in BBB permeability to small molecules [10,11] in rodents. Experiment one utilized an in vitro model of the blood–endothelial barrier (BEB) to assess whether CSD-like concentrations of K^+^ changed expression of HSP components in bEnd.3 brain endothelial cells; the endothelial cells comprise the first layer of the BBB [18]. bEnd.3 cells seeded in 6-well plates were treated with 60 mM KCl for 5 min (KCl pulse). aCSF were utilized as vehicle control. After treatment, the cell lysate was subjected to proteomic analysis. Using an unlabeled proteomics technique and targeted analysis, data indicate that a 5 min pulse of KCl (60 mM) did not change protein expression of HSP70, or the inducible form, HSP72 (Figure 1A). However, HSP90alpha, but not HSP90beta, was significantly increased after KCl stimulation as compared to aCSF control (Figure 1B). In addition, the HSP90 co-chaperone CDC37 and the endoplasmic reticulum isoform endoplasmin (i.e., GRP 94, gp96) were detected, with significant increases observed in endoplasmin levels after KCl (Figure 1C). Together, these data indicate that high extracellular K^+^ in the parenchyma can rapidly regulate HSP90 levels as well as endoplasmin in brain endothelial cells. These results justified further examination of the role of HSP90 in CSD-induced barrier dysfunction.

### 3.2. Inhibition of HSP90 Prevents Paracellular Breaches of the BEB/BBB

**Figure 2:**
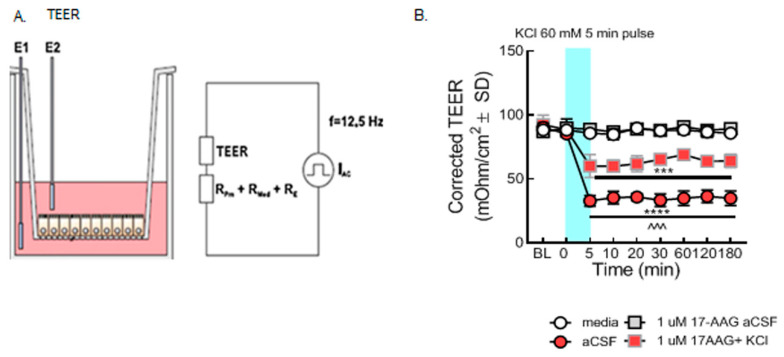
**Selective inhibition of HSP90 with 17-AAG mitigated the loss of barrier integrity caused by the KCl pulse in vitro.** bEnd.3 cells were cultured on transwell inserts in the presence of astrocyte-conditioned media on the abluminal side. The cells were treated with either 17-AAG (1µM) or vehicle (1% DMSO in media) 24 h before the KCl pulse. On the day of the experiment, the media on the abluminal side were changed to media containing 60 mM KCl (KCl pulse). Media containing aCSF were utilized as vehicle control. The medium was changed to a fresh one after 5 min KCl pulse. TEER measured before any treatment served as baseline. TEER was also assessed right before the KCl pulse (0 min), right after the KCl pulse (5 min), then 10, 20, 30, 60, 120, and 180 min after KCl/aCSF treatment. (**A**) Setup of the probe in transwell plates for measurement. (**B**) TEER values were significantly reduced after the KCl pulse (5 min, 60 mM) as compared to aCSF controls on the monolayer of bEnd.3 cells, suggesting loss of barrier integrity (KCl vs. aCSF: *** *p* < 0.001, **** *p* < 0.0001, as tested by one-way ANOVA with Tukey’s post-test). Twenty-four hour preincubation with 17-AAG (1 µM) attenuated the KCl-induced changes in TEER values (KCl + 17-AAG vs. KCl + vehicle: ^^^ *p* < 0.001, assessed by one-way ANOVA, Tukey’s post-test). Values were calculated as measured TEER—insert blank TEER avg and are reported as mean ± SD (*n* = 3 in triplicate).

**Figure 3:**
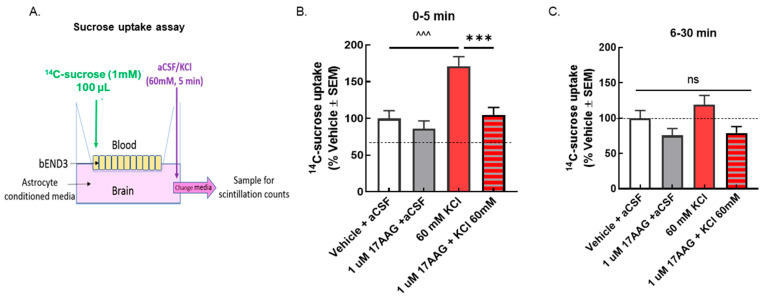
**Selective inhibition of HSP90 with 17-AAG reduced the elevated sucrose movement caused by the KCl pulse across the monolayer of bEnD.3 cells**. bEnd.3 cells were cultured in transwell inserts with astrocyte-conditioned media. The treatment was set as in the TEER experiments, 24 h pretreatment of 17-AAG (1 μM) or vehicle (1% DMSO in media), followed by KCl pulse (60 mM, 5 min). ^14^C-sucrose was added to the luminal side when KCl or aCSF was applied on the abluminal side. Samples were collected from the abluminal side after the 5 min KCl pulse (0–5 min) and 30 min after the KCl pulse (6–30 min) to detect radioactivity. (**A**) Setup of transwell paracellular leak model. (**B**) The amount of ^14^C-sucrose detected in the abluminal chamber was significantly increased after 5 min KCl exposure compared to aCSF controls, indicating reduced barrier integrity (KCl vs. aCSF at 5 min: ^^^ *p* < 0.001, as tested by one-way ANOVA with Tukey’s post-test). Treatment with the HSP90 inhibitor 17-AAG (1 μM, 24 h) prevented sucrose movement across the monolayer in KCl-treated cells, but it did not influence sucrose permeability in aCSF-treated cells (KCl + 17-AAG vs. KCl + vehicle: *** *p* < 0.001, assessed one-way ANOVA with Tukey’s post-test). (**C**) No significant difference among any groups was observed after 30 min. Values are normalized to percent of vehicle ± SEM (*n* = 4, dotted line).

Transendothelial electrical resistance (TEER), sucrose uptake, and in situ brain perfusion have been utilized in previous studies to assess barrier integrity [11,19]. The selective inhibitor of HSP90, 17-AAG, was used to pharmacologically assess the role of HSP90 in vitro and in vivo. In models of hypoxia and extreme insult, HSP90 has been implicated in BEB function by reorganizing tight junctions and adherens junctions [5,19,20,21,22]. Experiments two (Figure 2) and three (Figure 3) were designed to test global function by way of assessing paracellular permeability.

#### 3.2.1. 17-AAG Prevents Paracellular Leak Induced by KCl In Vitro

In the first experiment, paracellular integrity was assessed in the monolayer of bEnd.3 cells using TEER measurements after the KCl pulse in combination with HSP90 inhibitor. Our results revealed that the KCl pulse significantly reduced TEER values as compared to aCSF controls, indicating loss of BEB resistance as previously reported [12]. TEER reductions resulting from a 5 min KCl pulse were significantly prevented by preincubation with 17-AAG (1 µM), a concentration previously utilized in vitro [14] (Figure 2B). These data indicate that inhibition of HSP90 with 17-AAG restores integrity of the BEB after KCl exposure.

If the TEER reductions are functional, meaning a breach of paracellular integrity, an increase in ^14^C-sucrose movement across the BEB (luminal to abluminal, Figure 3A) would be expected after KCl and normalized after 17-AAG pretreatment. KCl exposure significantly increased the amount of ^14^C-sucrose detected in the abluminal chamber within the first 5 min after application (Figure 3B); this normalized by 30 min (Figure 3C). 17-AAG significantly reduced KCl-induced movement of sucrose across the bEnd.3 cell monolayer, without altering sucrose movement in those exposed to aCSF (Figure 3B), suggesting restoration of a paracellular integrity of the BEB in vitro.

**Figure 4:**
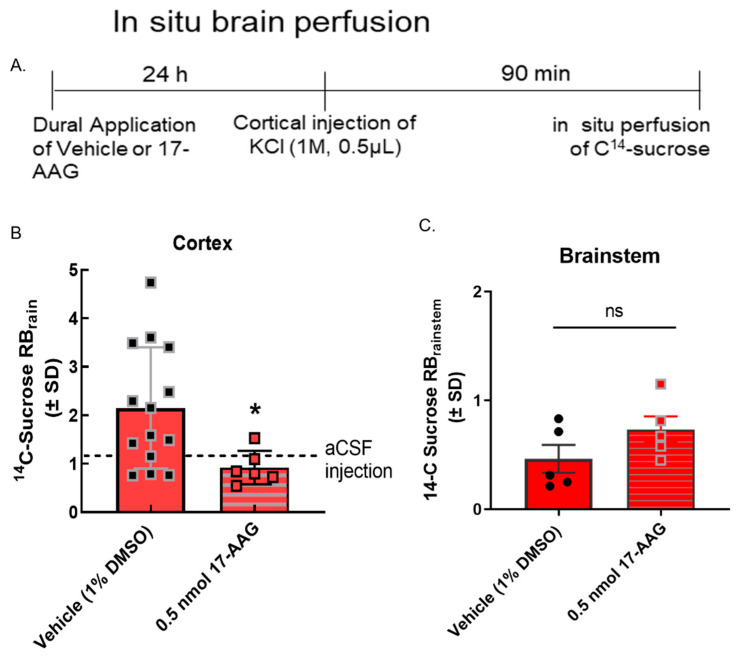
**17-AAG reduced cortical spreading depression-associated blood–brain barrier leak in vivo.** Dural cannulation was performed on female Sprague Dawley rats. After recovery, they were injected with 17-AAG (0.5 nmol) or vehicle (1% DMSO in saline) via dural cannula 24 h before CSD induction. CSD was induced by injection of KCl (1 M) through dural cannula. In situ brain perfusion was performed 90 min after the cortical injection of KCl. (**A**) Timeline of treatment, cortical injection, and perfusion. (**B**) ^14^C-sucrose uptake was measured in whole cortex and presented as the brain to plasma ratio (RBr) after 10 min brain perfusion. Dural application of 17-AAG (0.5 nmol) 24 h before cortical injections significantly reduced ^14^C-sucrose uptake in cortex as compared to vehicle control, suggesting that HSP90 inhibition could prevent KCl-caused BBB leak. (KCl + 17-AAG vs. KCl + vehicle: * *p* < 0.05, assessed unpaired *t*-test.) Dotted line represents the ^14^C-surose uptake measured in aCSF-treated animals. (**C**) No statistically significant difference was observed in sucrose uptake in the brainstem (*p* = 0.17). Values are mean ± SEM (*n* = 6–11). Circles and squares indicate individual subjects.

We have previously reported that BBB integrity is compromised during CSD events in preclinical models [11]. To determine if the inhibition of HSP90 reduced CSD-induced transient leak in vivo (i.e., intact BBB), in situ brain perfusion was performed at 90 min after the induction of CSD by cortical KCl injection. As previously reported [11], cortical injection of KCl significantly increased ^14^C-sucrose uptake into the parenchyma at 90 min (Figure 4B) suggesting paracellular leak. Pre-exposure to 17-AAG (0.5 nmol, in vivo dose established in [14]) significantly reduced sucrose uptake in the cortex at the same timepoint (Figure 4B) suggesting that in vivo, inhibition of HSP90 reduces paracellular breaches at the BBB.

#### 3.2.2. 17-AAG Increases Detection of Claudin 5 Total Protein after Cortical KCl Injection In Vivo

**Figure 5:**
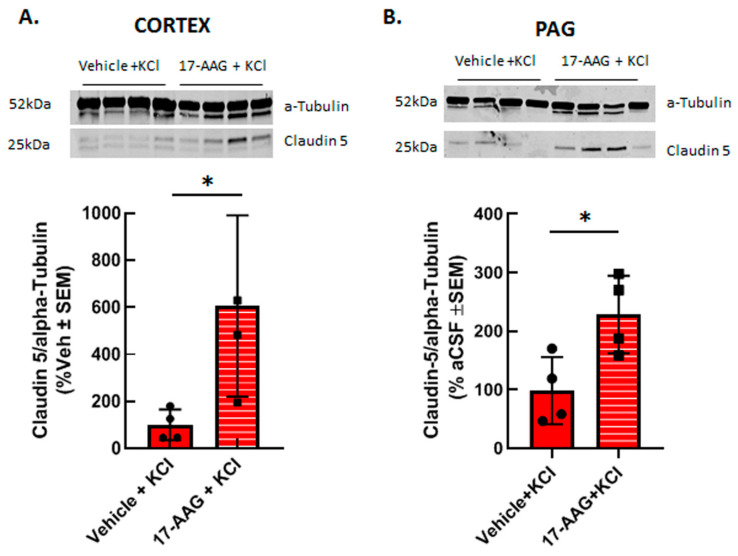
**17-AAG increased the expression of claudin 5 in cortex and PAG after induction of CSD.** Female Sprague Dawley rats underwent dural cannulation surgery. After recovery, 17-AAG (0.5 nmol) or vehicle (1%DMSO in saline) were injected via dural cannula 24 h before cortical KCl injection. Cortex and PAG tissue were harvested 90 min after CSD induction and subjected to Western immunoblotting. Representative images of cortex (**A**) and PAG (**B**) samples showing the expression of claudin 5 and α-tubulin, as loading control. Pretreatment of 17-AAG increased the expression of claudin 5 in cortex (**A**) and PAG (**B**) samples as compared to vehicle control. All data in panel **A** represent the % of vehicle-treated relative expression ± SEM (*n* = 4 in each group), whereas in panel **B,** all data represent the % of vehicle-aCSF pretreated controls’ relative expression ± SEM (*n* = 4/condition). * *p* < 0.05 as assessed by unpaired *t*-test. Circles and squares indicate individual subjects.

Previous reports have shown that HSP90 inhibition can restore tight junction proteins occludin and ZO-1 as well as matrix metalloproteases; to date claudin 5 has not been investigated. Therefore, we asked whether inhibition of HSP90 with 17-AAG changed expression of the tight junction protein claudin 5. Our previous studies using in vivo or in vitro techniques have indicated that cortical injection of KCl or KCl pulse in vitro does not change claudin 5 expression in cortical capillaries or endothelial cells as compared to naive or aCSF injected controls, respectively [11,23]. Therefore, cortical tissues from vehicle pretreated and 17AAG pretreated cortical tissues were compared. Application of 17-AAG 24 h prior to cortical KCl injection significantly increased the expression of claudin 5 in the cortex (Figure 5A) and in the PAG (Figure 5B) 90 min after KCl injection detected by Western immunoblotting. This data suggests that the beneficial effect of HSP90 inhibition observed in our in vitro experiments and in situ brain perfusion study may be related to increased expression of claudin 5.

### 3.3. Discussion

#### HSP90 Inhibition Improves BBB Integrity Showing Claudin 5 to Be Integral as a Neuroprotective Tight Junction Protein during and after CSD Events

**Figure 6:**
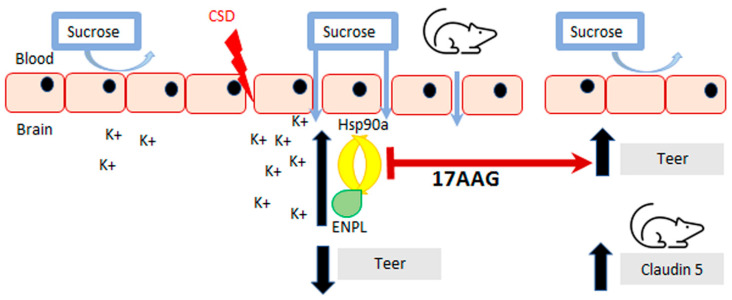
**Summary of findings**. **Inhibition of HSP90 using 17-AAG improves blood–brain barrier integrity.** Our results revealed that preincubation with 17-AAG mitigated the reduction of TEER values caused by the KCl pulse on the monolayer of bEnd.3 cells. The increased uptake of ^14^C-sucrose across the same endothelial monolayer induced by the KCl pulse was significantly attenuated after preincubation with the same HSP90 inhibitor. Pre-exposure to 17-AAG significantly reduced the transient BBB leak after CSD induced by cortical KCl injection as determined by in situ brain perfusion in female rats. The pharmacological blockade of HSP90 increased the detection of claudin 5 in cortex and PAG, which can play a part in the effect of HSP90 inhibition to protect BBB integrity.

Inhibition of HSP90 isoforms is a strategy being investigated for cancer, stroke, and pain, which are all associated with induction of cortical spreading depression events and breaches of BBB integrity [2,3,4]. Previous work by our group has demonstrated that CSD induction promotes transient paracellular opening of the BBB [11]. Using both an in vitro model, for the blood–endothelial barrier (BEB), and an in vivo model with a complete BBB, we now report that HSP90 inhibition with 17-AAG as a pretreatment before CSD induction restores BEB/BBB paracellular integrity as measured by TEER, sucrose uptake, and in situ brain perfusion. The inhibition of HSP90 also increased the detection of claudin 5 total protein which may contribute to its effect on BEB/BBB integrity. Figure 6 summarizes our findings. This novel observation supports the role of HSP90 in negatively regulating the integrity of the BBB and suggests the development of new therapeutic approaches targeting HSP90 in headache disorders.

BBB disruption leading to damage and increased paracellular permeability include hypoxic or inflammatory states associated with disease [24,25,26,27,28,29,30,31,32,33]. CSD has been shown to induce neuroinflammation as well as increase reactive oxygen species [3,34]. Studies have shown that HSP90 inhibition decreases inflammation in models of arthritis and stroke, such as MCAO [4,35,36], as well as reduces oxidative stress specifically in the microvasculature of the brain [20]. In addition, increased matrix metalloprotease-9 (MMP-9) expression can further disrupt barrier function allowing for decreased physical integrity [37]. CSD events have been documented to increase MMP-9 expression [10,34]. HSP90 inhibition decreased MMP-9 expression in a stroke model [36] as well as lowered inflammatory markers. This same group also showed that HSP90 inhibition prevented a reduction in TJ proteins occludin and ZO-1 [36]. Previously, we reported that CSD induction with cortical injection of KCl does not significantly change detection of TJ proteins (i.e., claudin 5) in cortex or brainstem as compared to aCSF controls [11]; however, KCl pulse decreases corrected total cell fluorescence of claudin 5 as compared to controls in bEnD.3 cells, but no significant changes in the surface levels of claudin 5 were observed after KCl treatment [23]. Our study now shows that pretreatment with HSP90 inhibitor 17-AAG increased expression of TJ protein claudin 5 in the cortex (site of paracellular leak) and periaqueductal grey (PAG, no paracellular leak observed). Increased expression of claudin 5 could add to the positive effects necessary for maintaining BBB integrity during and post-CSD. These align with the findings of Yaguchi and colleagues showing that use of HSP90 inhibitor 17-DMAG (17-dimethylaminoethylamino-17-demethoxygeldanamycin) in a middle cerebral artery occlusion model of stroke (MCAO) reduced the associative ischemic tissue damage and neurological function by normalizing MCAO increases in occludin and zona occludens-1 (ZO-1) [36]. Thus, inhibiting HSP90 may be a viable strategy to restore BBB function at the level of the tight junction during pathologies inducing paracellular permeability.

While the direct mechanisms by which HSP90 and claudin 5 interact are unknown, some studies suggest possible mechanisms of interaction. A recently published study suggests that hypoxia-induced injury of the BBB is reduced through regulation of claudin 5 by autophagy [38]. Their findings show that in hypoxia-induced damage in cells, breakdown of the BBB is partially caused by caveolin-1 (CAV1)-mediated redistribution of claudin 5 into the cytosol of brain microvascular endothelial cells (BMECs). When they induced autophagy in the cells and noted degradation of CAV1, there was less aggregation of claudin 5 in the cytosol of BMECs [38]. When autophagy was inhibited via genetic or chemical methods, this effect was reversed and claudin 5 aggregates formed in the cytosol leading to further BBB breakdown [38]. With this novel mechanism in mind, there has been evidence that HSP90 inhibitor 17-AAG induces the autophagic pathway [39,40] by stimulating recruitment of microtubule-associated proteins 1A/1B light chain 3B (LC3B) in vitro and in vivo. While these reports suggest one possible mechanism by which 17-AAG increased claudin 5 detection in the cortex and PAG, additional studies are required for full elucidation. Alternatively, HSP90s directly interact with ZO-1 to phosphorylate this anchoring protein for claudin 5 leading to ubiquitination and degradation [41,42,43] in a manner sensitive to 17-AAG. We have previously observed that high extracellular K^+^ promotes ZO-1 phosphorylation in bEnd.3 endothelial cells. Thus, 17-AAG may be preventing ZO-1 degradation to indirectly increase detection of claudin 5 [23].

Study Limitations and Alternative interpretations. While our findings shed light on the role that HSP90 plays in BBB maintenance, some limitations should be noted. In our previous work, we showed that detection of claudin 5 total protein did not change after CSD induction [11], but rather inducted relocalization [23]. Here, we report that 17-AAG increased total claudin 5 detection; thus, two different mechanisms may be at play. In addition, some variability was noted in claudin expression within treatments; as female rats were used, but estrous stage not determined, this may reflect estrogen regulation of claudin 5, independent of CSD and 17-AAG [44]; investigating these effects are warranted. Third, 17-AAG is a pan-HSP90 inhibitor. Given the data herein showing KCl increased endoplasmin levels in bEND.3 endothelial cells and one report that 17-AAG has similar affinity to this protein [45], use of a selective endoplasmin (GRP94, gp96) inhibitor is justified [46]. Lastly, HSP90 has more than 200 documented client proteins that may be implicated in our observed results, further supporting more research into these HSP90–claudin 5 interactions [47].

## 4. Conclusions

Future studies on BBB dysregulation are required to limit the influence CSD events may have on chance of stroke in patients experiencing migraine with aura. Claudin 5 has been suggested to be a prominent candidate in BBB maintenance and regulation to treat ischemic stroke [48] and may be a viable target for preventative treatment in people who experience spreading depression. Current data suggest that inhibition of HSP90 with the selective agent 17-AAG reduces CSD-associated BBB paracellular leak at the endothelial cell level both in vitro and in vivo via increasing claudin 5 expression. Investigation of cellular and molecular mechanisms underlying this effect is warranted and requires future studies to develop new therapeutic approaches targeting HSPs as a treatment for stroke, headache/migraine, and other diseases where an impaired BBB is implicated.

## Data Availability

All data generated or analyzed during this study are available from the corresponding author on reasonable request. The whole data set of proteomic analysis is available in Wahl et al. (2022) [23].

## Abbreviations

BBB	blood–brain barrier
BEB	blood–endothelial barrier
CSD	cortical spreading depression
HSP	heat shock protein
TEER	trans endothelial electric resistance
17-AAG	17-allylamino-17-demethoxy-geldanamycin
